# Family *Herpesviridae* and neuroinfections: current status and research in progress

**DOI:** 10.1590/0074-02760220200

**Published:** 2022-11-21

**Authors:** Vanessa Cristine de Souza Carneiro, Jéssica Gonçalves Pereira, Vanessa Salete de Paula

**Affiliations:** 1 Fundação Oswaldo Cruz-Fiocruz, Instituto Oswaldo Cruz, Laboratório de Virologia Molecular, Rio de Janeiro, RJ, Brasil

**Keywords:** herpesvirus, neuroinfection, neurodegeneration

## Abstract

This article addresses the relationship between human herpesviruses (HHVs) and neuroinfections. Alphaherpesviruses, betaherpesviruses and gammaherpesviruses are neurotropic viruses that establish latency and exhibit reactivation capacity. Encephalitis and meningitis are common in cases of HHV. The condition promoted by HHV infection is a purported trigger for certain neurodegenerative diseases. Ongoing studies have identified an association between HSV-1 and the occurrence of Alzheimer’s disease, multiple sclerosis and infections by HHV-6 and Epstein-Barr virus. In this review, we highlight the importance of research investigating the role of herpesviruses in the pathogenesis of diseases that affect the nervous system and describe other studies in progress.

Neurological disorders are among the leading causes of global mortality. Although the aetiology of neuroinfections is unclear, recent studies have linked the onset of neurological disorders to herpesvirus infection, which can cause neurological symptoms or lead to immune responses that trigger pathological signs. Currently, this relationship is primarily based on epidemiological data regarding infections and the seroprevalence of patients with neurological disorders.

The *Herpesviridae* family comprises nine viruses that cause infections in humans and is divided into three subfamilies: *Alphaherpesvirinae*, *Betaherpesvirinae* and *Gammaherpesvirinae* [International Committee on Taxonomy of Viruses (ICTV 2021)]. These viruses are prevalent worldwide and cause various diseases, including cold sores, genital herpes, stromal keratitis, cancer, meningitis and encephalitis. All herpesviruses have two replication cycles: lytic and latent. Lytic replication produces particles to infect other cells and organisms, whereas latency has limited gene expression and absence of infectious particles. Herpesviruses establish latency at different sites (Table) and can cause disease during both primary infection and reactivation; however, the mechanisms leading to latency and reactivation and the viral and host factors controlling them remain unclear. Thus, we present the main clinical manifestations of each type of herpesvirus and demonstrate that, in addition to classical disorders such as encephalitis, other neurological manifestations have been associated with herpesviruses, such as multiple sclerosis (MS), epilepsy, schizophrenia and Alzheimer’s disease (AD).

**TABLE:** Family *Herpesviridae* and neuroinfections

	HSV-1	HSV-2	VZV	EBV	HCMV	HHV-6A	HHV-6B	HHV-7	HHV-8
Year of discovery	1920^1^	1920^1^	1952^17^	1964^71^	1956^26^	1986^40^	1988^41^	1990^60^	1994^80^
Name	Herpes simplex virus 1	Herpes simplex virus 2	Varicella-zoster virus	Epstein-Barr virus	Human cytomegalovirus	Human herpesvirus-6A	Human herpesvirus-6B	Human herpesvirus-7	Human herpesvirus-8
Taxonomy ICTV (2021)	Human alphaherpesvirus 1	Human alphaherpesvirus 2	Human alphaherpesvirus 3	Human gammaherpesvirus 4	Human betaherpesvirus 5	Human betaherpesvirus 6A	Human betaherpesvirus 6B	Human betaherpesvirus 7	Human gammaherpesvirus 8
Discoverer(s)	Gruter^1^	Gruter^1^	Thomas H. Weller^17^	Michael Anthony Epstein^71^	Weller Smith and Rowe^26^	Salahuddin^40^	Yamanishi^41^	Frenkel^60^	Yuan Chang^80^
Genome	152 kb^3^	155 kb^4^	125 kb^18^	170 kb^70^	236 kb^27^	159 kb^43^	162 kb^44^	144 kb^61^	140 kb^82^
Latency	Neurons^5^ ^,^ ^7^	Neurons^5^ ^,^ ^7^	Neurons^20^	Memory B cells^73^	Renal epithelial cells and salivary glands^31^	Monocytes, macrophage and glial cells ^(^ ^48^ ^,^ ^50^	Salivary glands, monocytes, macrophages and glial cells^47^ ^,^ ^48^ ^,^ ^50^	Unclear	B lymphocytes^83^ ^,^ ^84^
Clinical manifestations	Skin lesions^5^	Genital or anal blisters or ulcers^6^	Varicella, herpes zoster^19^ ^,^ ^21^	Infectious mononucleosis^72^	Congenital viral infection^34^	Asymptomatic^45^ ^,^ ^49^ ^,^ ^56^	Exanthema subitum, present in infantile roseola^42^ ^,^ ^45^ ^,^ ^49^ ^,^ ^56^	Exanthema subitum, fever without exanthema, febrile convulsions and status epilepticus^65^	Kaposi’s sarcoma, primary effusion lymphoma and multicentric Castleman disease^81^
Neurological manifestations	Encephalitis, meningitis, and meningoencephalitis ^10^ ^-^ ^12^	Encephalitis, meningitis, and meningoencephalitis^10^ ^-^ ^11^	Post-herpetic neuralgia, meningoencefalitis, ischemic stroke, aneurysm and cerebral venous thrombosis^21^ ^-^ ^23^	Meningitis, encephalitis, myelitis, psychoses and “Alice in Wonderland” syndrome^72^ ^,^ ^75^ ^,^ ^76^	Encephalitis and neurodevelopmental deficits^35^ ^,^ ^36^ ^-^ ^38^	Encephalitis and multiple sclerosis^49^ ^-^ ^51^ ^,^ ^56^ ^,^ ^58^	Seizures, encephalitis, mesial temporal lobe epilepsy and multiple sclerosis^49^ ^-^ ^54^	Seizures, encephalitis, meningoencephalitis, facial palsy, vestibular neuritis, severe headache, drowsiness, fatigue, photosensitivity, ataxia and coma^69^	Under investigation
Research in progress	Alzheimer’s disease^14^ ^-^ ^16^	Alzheimer’s disease^14^ ^-^ ^16^	-	Multiple sclerosis^74^ ^,^ ^77^ ^,^ ^78^	Schizophrenia^37^ ^-^ ^39^	Multiple sclerosis^56^ ^,^ ^59^	Epilepsy and multiple sclerosis^48^ ^,^ ^53^ ^,^ ^54^ ^,^ ^59^	Epilepsy^66^	_

EBV: Epstein-Barr virus; HCMV: human cytomegalovirus; HHV: human herpesvirus; HSV: herpes simplex virus; ICT: International Committee on Taxonomy of Viruses; VZV: varicella zoster virus.

Alphaherpesvirus Human alphaherpesvirus 1 and 2 Herpes simplex viruses (HSVs) 1 and 2

HSV, which belongs to the *Alphaherpesvirinae* subfamily, was first isolated in 1920 by Gruter;^1^ however, HSV categorisation into herpes simplex 1 (HSV-1) and herpes simplex 2 (HSV-2), based on epidemiological, clinical and immunological differences, was done only in 1968.^2^ ^)^ HSV has a double-stranded DNA genome, approximately 152 kb in HSV-1 and 155 kb in HSV-2.^3^ ^,^ ^4^ ^)^ In general, HSV-1 causes skin lesions in the orolabial region; however, it can also cause serious complications such as herpetic keratitis.^5^ ^)^ HSV-1 can also cause genital herpes, although this is a characteristic symptom of HSV-2 infection, with genital or anal blisters or ulcers.^6^

After infection and replication in epithelial cells, HSV migrates via retrograde axonal transport to the dorsal root ganglia, where it establishes latency in neurons and can cause neurological disorders.^5^ ^,^ ^7^ ^)^ After primary infection, immunological changes in the host organism can lead to HSV reactivation in which viral particles are transported to axon terminals in an anterograde fashion.^8^ Released viral particles promote the infection of new epithelial cells and can infect neurons and reach the central nervous system (CNS), triggering inflammatory responses driven by microglia. This persistent activation has harmful effects on neurons and contributes to neurological diseases.^9^

HSV is widely associated with neurological disorders, such as encephalitis, meningitis and meningoencephalitis,^10^ ^)^ where immunosuppression by other etiological agents during the course of infection is one of the triggers for the reactivation of HSV-1 and HSV-2, worsening the prognosis.^11^ ^)^ In fact, a recent study observed the occurrence of HSV encephalitis in post-coronavirus disease (COVID) syndrome patients, possibly caused by reactivation during immune dysregulation in SARS-CoV-2 infection.^12^

Several studies have proposed an association between HSV-1 infection and neurodegenerative disease.^8^ ^,^ ^13^ ^)^ It is hypothesised that HSV-1 modulates neuronal apoptosis during acute and latent infection, which may be related to changes in neuronal processes, leading to neuronal damage and brain diseases.^8^ ^)^ An increasing number of studies support the involvement of HSV-1 in AD. Viral infection induces the accumulation of beta-amyloid proteins and phosphorylated tau protein, which are considered important components in AD progression.^14^ ^-^ ^16^ ^)^ Despite many questions that require clarification, these data indicate a strong link between HSV-1 and AD, warranting further investigation.

Human alphaherpesvirus 3 Varicella zoster virus (VZV)

VZV, isolated in 1952 by Thomas H. Weller in tissue culture, has a genome of 125 kb^(17, 18)^ and infects epithelial cells of the upper respiratory mucosa, subsequently causing a vesicular eruption that is widely distributed throughout the body and varicella.^19^ Similar to other alphaherpesviruses, it infects neurons during primary infection, reaching the ganglia by retrograde axonal transport where latency is established.^20^ ^)^ Decline in immunity can reactivate VZV, causing herpes zoster and triggering complications such as neuropathic pain called post-herpetic neuralgia, resulting from neuron damage caused by the inflammatory response to the reactivation and migration of VZV.^21^ Thus, similar to other herpesviruses, VZV also causes meningoencephalitis as a consequence of viral reactivation.^22^ In addition, VZV penetrates the walls of cerebral arteries after reactivation in nerve ganglia and can cause vasculopathy via productive infection by the virus in the cerebral arteries, leading to ischemic stroke, aneurysm and cerebral venous thrombosis.^22^ ^,^ ^23^ ^)^ Studies have indicated that neuroinflammation and immunological changes triggered by some neurotropic viruses play an important role in neurodegeneration. Researchers have also aimed to identify a link between VZV infection and neurodegenerative diseases.^24^ ^,^ ^25^

Betaherpesviruses Human betaherpesvirus 5 Human cytomegalovirus (HCMV)

HCMV, first isolated in 1956,^26^ ^)^ has the largest genome of any known human virus. Its genome is 236 kb in size and contains a double-stranded DNA molecule comprising two unique regions, each flanked by inverted repeats.^27^ HCMV is a β-herpesvirus found at a frequency of 40-100% in the adult population worldwide.^28^ ^)^ Being a herpesvirus, it establishes latency that can lead to periodic reactivation. Infections in healthy individuals are commonly asymptomatic;^29^ ^)^ however, immunocompromised individuals can develop serious illnesses and even die because of HCMV infection.

Although the primary infection in immunocompetent adults is asymptomatic, viremia can persist for weeks or even months.^30^ ^)^ HCMV can infect most cells in the human body, and infection of the renal epithelial cells and salivary glands allows viral transmission through saliva and urine for months, thereby transmitting it to new hosts. When the host immune response fails, the HCMV may access the CNS through the “Trojan horse” system by infecting endothelial cells, monocytes or macrophages. Some studies have also suggested its propagation through cerebrospinal fluid (CSF).^31^

Tissue necrosis observed in the CNS is because of direct cytopathology mediated by HCMV as well as by cytotoxic cytokines, interleukins and tumour necrosis factor.^32^ ^)^ Most pre- or immediately post-natal infections are characterised by increased viral replication, greater risk of persistence and, consequently, more severe conditions than those associated with infections acquired at an older age.^33^ ^)^ HCMV is the leading cause of congenital viral infections, the most common non-genetic cause of hearing loss and a major cause of neurodevelopmental delay. It affects 0.2-2% of all newborns worldwide.^34^ ^)^ Approximately 10-15% of children with congenital HCMV are symptomatic at birth. The infection presents with various symptoms, such as intrauterine growth retardation, hepatosplenomegaly, jaundice and neurodevelopmental deficits.^35^

In adults, HCMV encephalitis presents with several neurological symptoms, most of which occur in human immunodeficiency virus (HIV)-infected and immunosuppressed patients.^36^ It has an observable affinity with the limbic system, an area known to be affected in schizophrenia, and is a chronic neuropsychiatric disorder characterised by abnormalities involving brain structure and function.^37^

An immunocompetent patient who presented with auditory hallucinations, delusions, tangential thinking and flattened affect was initially diagnosed with schizophrenia until a post-mortem analysis based on neuropathological findings and CSF antibody levels indicated HCMV encephalitis.^38^ ^)^ An association with a sensorimotor control deficit has been identified in rodent models infected with HCMV, similar to that reported in individuals with schizophrenia.^39^

Based on the literature, neuropathogenesis in individuals infected with HCMV is unclear because of the lack of an adequate experimental models and lack of dedicated research.

Human betaherpesvirus 6 Human herpesvirus 6 (HHV-6A/B) 

HHV-6 was first isolated in 1986 by Salahuddin et al. ^(^ ^40^ ^)^ in patients with lymphoproliferative diseases or acquired immunodeficiency syndrome. Subsequent studies suggested the existence of two variants of HHV-6, variant A or HHV-6A, initially isolated by Salahuddin et al.,^40^ and variant B or HHV-6B, which was isolated from peripheral blood lymphocytes of patients with sudden rash by Yamanishi et al.^41^ ^)^ The IVTV has recognised HHV-6 variants as two distinct viruses: HHV-6A, having a higher level of virulence and considered the most cytolytic, and HHV-6B, the causative agent of exanthema subitum, present in infantile roseola (an acute childhood disease that characteristically presents with a high fever followed by a generalised rash).^42^ However, the term HHV-6 remains in use to collectively refer to both species. Although the genomes of the two viruses share 90% global identity, the size of the HHV-6A genome is 159 kb, whereas that of HHV-6B is 162 kb.^43^ ^,^ ^44^ ^)^ HHV-6B was designated as a human B lymphotropic virus because it has tropism for B cells.^40^ HHV-6 is pleiotropic, replicates well in CD4+ T lymphocytes and can proliferate in macrophages, fibroblasts and other cells.^45^

HHV-6B infection is very common in children between the ages of 2 and 3 years.^46^ ^)^ It is transmitted horizontally via saliva, as the salivary glands function as a valuable reservoir for this virus.^47^ ^)^ However, the common latency site for HHV-6A and HHV-6B is the monocyte/macrophage cell population.^48^ ^)^ HHV-6A and HHV-6B can also infect the CNS and cause neurological disorders.^49^

Studies have reported that 95% of adults are seropositive for HHV-6. Other manifestations related to primary HHV-6 infection have already been investigated, and current research is focused on its neurotropic properties, suggesting a possible link with encephalitis, seizure disorders, AD and MS.^45^

Active or latent infection by HHV-6 in immune and glial cells can alter the sensitive balance between demyelination and remyelination, a process that defines the progression of MS. Although HHV-6 infection alone cannot trigger the onset of MS, it can worsen the inflammatory state of the CNS and exacerbate demyelination in these patients.^50^ The possible role of latent HHV-6 infection has also been considered, for no studies have reported evidence of active replication of viral particles in demyelinating MS lesions.^51^ ^)^ Moreover, permeabilisation of the blood-brain barrier (BBB) may facilitate the entry of HHV-6 into the CNS, as it has high tropism for activated CD4+ T lymphocytes.^50^

HHV-6 has also been associated with acute seizures and epilepsy in children.^52^ ^,^ ^53^ ^)^ Symptoms described in the context of primary infection and reactivation include febrile seizures, acute symptomatic seizures following encephalitis, status epilepticus and temporal lobe epilepsy.^54^ A significant moderately positive correlation was identified between A delta and C nerve fibre damage severity and HHV-6 infection in fibromyalgia, which is a disease in which patients experience chronic pain.^55^ ^)^ Owing to the high frequency of HHV-6 infection in the population, further investigations are essential.

The high degree of homology between HHV-6A and HHV-6B hinders the investigation of these viruses separately. However, primary HHV-6B infection results in roseola, whereas information concerning the clinical manifestations of primary HHV-6A infection is limited.^56^ ^)^ Although both viruses are neurotropic and cause neurological disorders such as encephalitis, studies have reported that HHV-6A exhibits greater neurovirulence.^57^ ^,^ ^58^ The roles of HHV-6A and HHV-6B have been investigated in the pathogenesis of MS; however, studies have reported a higher prevalence of HHV-6A in patients with MS.^59^

Human betaherpesvirus 7 Human herpesvirus 7 (HHV-7)

HHV-7, first isolated in 1990,^60^ ^)^ is closely related to HHV-6 and also has a double-stranded DNA genome of approximately 144 kb.^61^ ^)^ On comparison, we observed that HHV-6 is the most described and investigated in the literature, which justifies the need for further investigations regarding HHV-7.

HHV-7 infects CD4+ T lymphocytes and, less frequently, CD8+ and immature T cells.^62^ ^)^ Similar to other herpesviruses, it becomes established in the host after primary infection for prolonged periods, alternating between latent and lytic phases.^63^ However, the site of latency remains unclear.^60^ HHV-7 is ubiquitous and primary infection occurs primarily during infancy between the ages of 1 and 3 years, slightly later than HHV-6. By the age of 5 years, approximately 90% of the population is infected with HHV-7.^64^ ^)^ HHV-7 infection has different clinical presentations in children, similar to HHV-6, such as exanthema subitum, fever without exanthema, febrile convulsions and status epilepticus^65^. Till date, available data support a possible association between HHV-7 and epilepsy.^66^ ^)^ Recent evidence has suggested that inflammation plays a role in epileptogenesis.^67^

Information regarding how HHV-7 crosses the BBB and causes invasion of the CNS is scarce.^68^ Recently, a small number cases of HHV-7-related encephalitis or encephalopathy have been described in children, adults and immunocompetent or immunocompromised patients.^69^ The clinical presentation of CNS involvement may be excessively heterogeneous to distinguish it from other neurological disorders, including fever, seizures, encephalitis, meningoencephalitis, facial palsy, vestibular neuritis, severe headache, drowsiness, fatigue, nausea, vomiting, photosensitivity, ataxia and coma.^69^

Gammaherpesviruses Human gammaherpesvirus 4 Epstein-Barr virus (EBV)

EBV is a gammaherpesvirus with a DNA genome of approximately 170 kb.^70^ ^)^ It was identified in 1964 by Michael Anthony Epstein in isolates from patients with Burkitt lymphoma.^71^ EBV infection in adults leads to infectious mononucleosis; however, it can also be associated with other complications, in addition to being associated with malignancies.^72^ ^)^ EBV infects epithelial and memory B cells, where it establishes latency,^73^ ^)^ and can replicate in the CNS and disrupt the integrity of the BBB.^74^ EBV infection is associated with neurological disorders, such as meningitis, encephalitis, myelitis, psychoses and ‘’Alice in Wonderland’’ syndrome, among others, which can affect even immunocompetent individuals both during primary infection and during reactivation.^72^ ^,^ ^75^ ^,^ ^76^ ^)^ Some studies have indicated an association between EBV infection and neurodegenerative diseases, and strong evidence suggests a role of EBV infection in the pathogenesis of MS.^74^ ^)^ MS is an immune-mediated disease characterised by permanent inflammation of the CNS, causing loss of neural tissue. Hence, viral infection may be a crucial factor in the development and progression of MS. Several researchers have theorised that EBV may contribute to the spread of inflammation and injury in the CNS, triggering an immune response that is important in the pathogenesis of MS.^77^ ^)^ A longitudinal study has revealed the risk of MS after EBV infection in addition to the increased concentrations of the neurofilament light chain, a biomarker of neurodegeneration, after EBV infection.^78^ ^)^ These data indicate the important role of EBV infection in the pathogenesis of MS.

Research in “long COVID” patients has identified EBV reactivation, suggesting that its symptoms, including neurological manifestations, may be the result of EBV reactivation induced by SARS-CoV-2 infection.^79^ Studies such as this emphasise the importance of investigating herpesvirus infections in the context of COVID-19.

Human gammaherpesvirus 8 Human herpesvirus 8 (HHV-8) 

HHV-8 was identified in 1994 by Yuan Chang^80^ ^)^ as the causative agent of Kaposi’s sarcoma in patients with acquired immunodeficiency syndrome. Subsequent studies have strongly associated this herpesvirus with other pathologies such as primary effusion lymphoma and multicentric Castleman disease in immunodeficient individuals; however, HHV-8 can also affect immunocompetent individuals.^81^ ^)^ HHV-8 contains a double-stranded DNA genome of approximately 140 kb.^82^ ^)^ It exhibits tropism for endothelial, epithelial and lymphocyte cells and establishes latency in B lymphocytes.^83^ ^,^ ^84^ ^)^ To date, knowledge regarding the ability of HHV-8 to infect neural cells and cause neurological disorders is limited. However, research has demonstrated the ability of HHV-8 to infect neural cells and act as a reservoir for latent infection.^83^ ^,^ ^85^ ^)^ Studies have investigated the association between neurological disorders and HHV-8 infection, including amyotrophic lateral sclerosis and MS.^86^ ^,^ ^87^ Although the detection of HHV-8 DNA in brain tissues of MS patients indicates neurotropism, a possible association between HHV-8 infection and MS pathogenesis needs to be further investigated.^87^ ^)^ These findings provide novel insights into the association of HHV-8 with neuronal diseases and require further clarification.

Herpesvirus in the context of neuronal disorders

Herpesviruses have been strongly associated with neurological disorders, with meningitis and encephalitis being triggered by all HHVs except HHV-8.^10^ ^,^ ^49^ ^,^ ^69^ ^)^ HCMV is associated with neurodevelopmental deficits and schizophrenia.^38^ ^,^ ^39^ VZV has been implicated in cases of vasculopathy leading to ischemic stroke, aneurysm and cerebral venous thrombosis.^22^ ^,^ ^23^ EBV has been related to psychosis and distortion of perception, such as the Alice in Wonderland syndrome.^72^ ^,^ ^75^ ^,^ ^76^ HHV-6 and HHV-7 are considered as possible causes of epilepsy.^53^ ^,^ ^54^ ^,^ ^66^ ^)^ Although HHV-8 is not known to be associated with neurological diseases, several studies have indicated such a relationship; future research may elucidate new findings regarding this association.^85^ Ongoing studies surrounding the relationship between herpesvirus infection in neurodegenerative diseases are worth mentioning, such as the association between AD and HVS and the relationship between MS and EBV and HHV-6 infection (Table).^15^ ^,^ ^50^ ^,^ ^51^ ^,^ ^78^ In recent years, some studies have investigated a possible association between alpha herpesvirus infections and Parkinson’s disease.^13^ ^,^ ^25^

Nonetheless, several issues need to be clarified, ranging from stresses that can induce recurrent virus reactivation to viral particles produced after reactivation that can reach the CNS, causing diffuse acute infection and/or neurological manifestations. Recently, the reactivation viral has been correlated with the production and accumulation of neuropathological biomarkers of AD.^88^ The different cellular and molecular mechanisms underlying the acute and long-term damage caused by herpesvirus infection in the CNS should be investigated to bridge these gaps in knowledge.

It is worth noting that although herpesviruses are highly prevalent and are latently present in all infected individuals, not everyone will experience reactivation or develop neurological manifestations. The fact that certain individuals are more likely than others to develop serious diseases and neurological manifestations after herpes infection can be partially explained by the existence of genetic polymorphisms in humans and the intrinsic, innate and adaptive immune responses that are fundamental for controlling diseases and infections caused by herpesviruses.^89^

Although the number of studies exploring the mechanisms of action by which viral infections can directly or indirectly contribute to the development of neurological disorders has increased over the years, these studies remain insufficient.^90^

With the advancement in technologies and tools for the investigation of virological, genetic and immunological factors, several knowledge gaps can be addressed.

To summarise, understanding the pathogenesis of these diseases and exploring new theories may facilitate the development of new diagnostic and therapeutic strategies. Reviewing and updating evidence regarding the relationship between viral infection and neurological disorders are essential to confirm the hypotheses and potential of herpesviruses as triggering agents of neurological disorders.
